# Case Report: A rare case of prosthetic valve infective endocarditis caused by *Aerococcus urinae*

**DOI:** 10.12688/f1000research.12776.3

**Published:** 2018-03-28

**Authors:** Muhammad Adeel, Saman Tariq, Hisham Akthar, Ahmed Zaghloul, Corina Iorgoveanu, Carina Dehner

**Affiliations:** 1 Yale New Haven Health, Bridgeport Hospital, Bridgeport, CT, USA; 2 Galway University Hospital, Galway, Ireland; 3 University of Connecticut Health Center, Farmington, CT, USA; 4 School of Medicine, Yale University, New Haven, CT, USA

**Keywords:** infective endocarditis, prosthetic valve endocarditis, Aerococcus urinae

## Abstract

Infective endocarditis (IE) is a serious and life-threatening cardiac condition, most commonly caused by staphylococci, Streptococcus viridans, and enterococci. However, in special settings, IE can be caused by rare organisms. Here we present a case of IE caused by *Aerococcus urinae* in a 75-year-old man with a bioprosthetic aortic valve. *Aerococcus* *urinae* is a gram-positive, catalase-negative microorganism and is usually an isolate of complicated urinary tract infections in the elderly male population.  Improvements in diagnostic testing including use of matrix-assisted laser desorption ionization– a time of flight mass spectrometry (MALDI-TOF MS) have played an important role in recognition of *Aerococcus urinae.*

## Introduction

IE is a serious and potentially life-threatening condition. Expedite recognition, diagnosis, and treatment is critical. The diagnosis of IE is based on Dukes criteria or its modifications ^1^. Risk factors for IE include advanced age (> 60 years), male gender, history of intravenous drug use, poor dentition, structural or valvular heart disease and presence of prosthesis. Here, we describe a rare case of IE caused by *Aerococcus urinae*, a gram-positive, catalase-negative coccus that grows in clusters. *Aerococcus urinae* is a rare organism and since its first reported in 1967 ^2^, has been increasingly recognized as a causative pathogen of urinary tract infections and rarely IE. In the past, reported cases showed poor outcome; however recent Swedish epidemiological study reported the favorable outcome ^3^.

## Case report

A 75-year-old Caucasian man presented to his local hospital with malaise, fever, and nausea for five days. He had a bio prosthetic aortic valve replacement for mixed aortic valve disease 12 years ago; further significant past medical history included placement of a permanent pacemaker for complete heart block, right total hip replacement, hypertension and benign prostatic hyperplasia (BPH). The patient had no history of smoking, alcohol consumption or illicit drug use. The patient had no recent surgeries or dental work, and the review of systems was unremarkable. The physical exam revealed vital parameters of HR 97 bpm regular, BP 134/87, the temperature of 101.5°F, respiratory rate of 18 per minute and oxygen saturation of 96% on room air. On precordial auscultation, a systolic and a diastolic murmur were heard in the aortic area, mild bi-basal crackles, but no jugular venous distention or peripheral edema. The rest of the physical exam was unremarkable. The labs showed a normal white cell count (WCC) of 9.9 × 10 ^6^/L, elevated C-reactive protein to 214.9 mg/L (normal <5 mg/l) and a hemoglobin of 11.2 g/dl), the other labs were unremarkable. His mid-stream urine showed WCC < 20; red cell count (RCC) of 20–50 and it grew mixed organisms, all considered part of the normal flora. Chest X-ray, CT scan of the brain, thorax, abdomen, and pelvis did not show any source of infection.

The patient was empirically commenced on IV piperacillin-tazobactam and vancomycin. Blood cultures collected at the time of admission grew *Aerococcus urinae* in both bottles. A repeat set of blood cultures corresponding to a spike of fever in the following 24 hours also grew *Aerococcus urinae* in both bottles; all cultures were sensitive to ampicillin (MIC 0.064 mg/L) and gentamicin (MIC 2 mg/L).

A trans-thoracic echocardiogram showed mild aortic regurgitation and mitral regurgitation with no clear vegetation, however, trans-esophageal echocardiogram (TOE) showed normal left ventricular function with moderate aortic regurgitation due to large mobile vegetation on the bio-prosthetic aortic valve. There was no peri-valvular abscess or features of the paravalvular abscess noted (See Image 1a and 1b). Pacemaker lead and right-sided valves were not involved.

**Figure 1.**
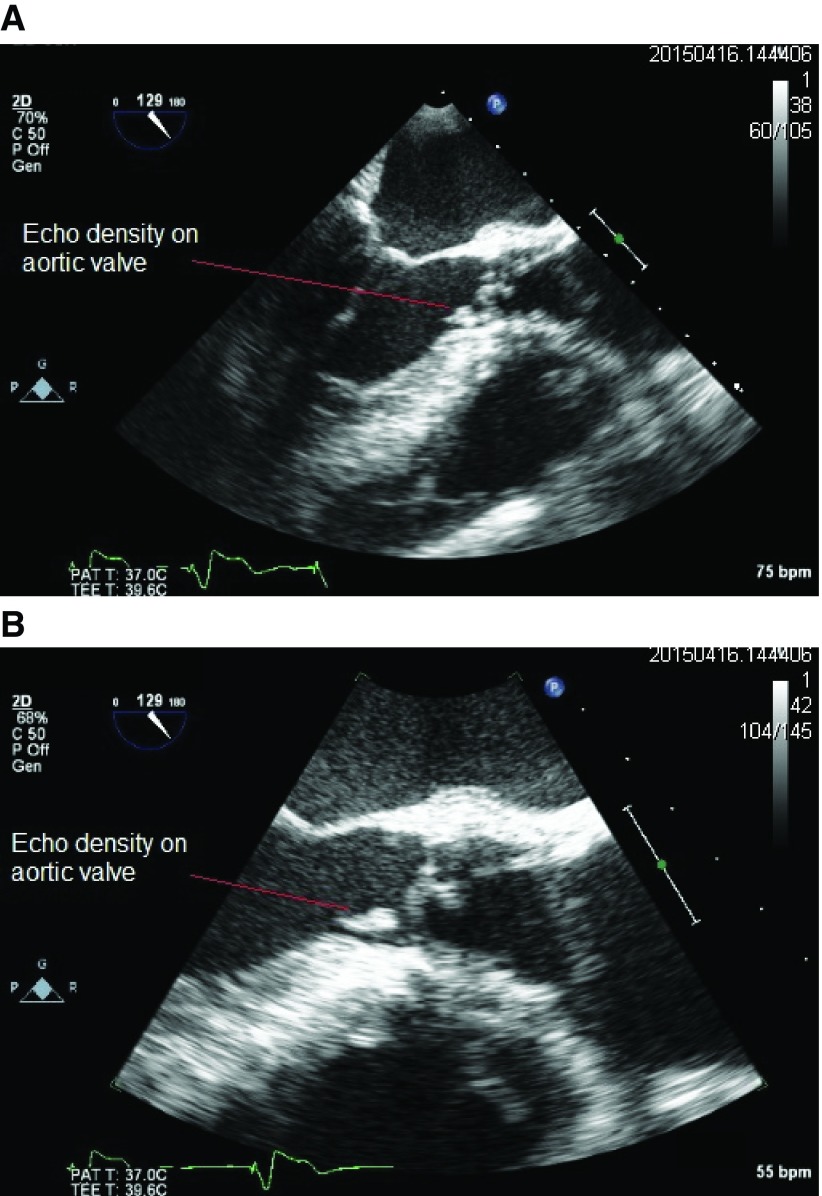
**1A**: Transesophageal echocardiogram (TEE), mid-esophageal view showing mobile echo density on the prosthetic aortic valve. **1B**: Transesophageal echocardiogram (TEE), mid-esophageal view enlarged to show mobile echo density on the prosthetic aortic valve.

Clinical presentation, echocardiographic findings, and positive blood cultures fulfilled Duke’s criteria (Hoen *et al.*, 1996) for IE. The patient was managed as prosthetic aortic valve endocarditis from *Aerococcus urinae* with IV amoxicillin 2 grams every 4 hours, and gentamicin 1 mg/kg twice daily as per hospital guidelines for IE. IV antibiotic therapy for six weeks in total with possible surgery for prosthetic valve replacement was planned (Truninger *et al.*, 1999).

Despite prompt initiation of appropriate antibiotic treatment and intensive clinical monitoring, the patient failed to improve this hospitalization and developed sudden pulmonary edema and worsening aortic regurgitation on repeat transthoracic echo and unfortunately died due to rapid deterioration before surgery. As per family’s wishes, an autopsy was not performed.

## Discussion

*Aerococcus urinae* is a gram-positive, catalase-negative coccus which grows in clusters. It is mostly associated with urinary tract infections in elderly men, especially in the setting of structural abnormalities, e.g. BPH, urethral strictures and nephrolithiasis. It has been associated with culture-negative infective endocarditis ^4^. It is reported to be sensitive to penicillins/cephalosporins and resistant to sulfonamides and aminoglycosides ^5^. By now, more than 40 cases of IE caused by Aerococcus urinae have been reported ^6^ likely due to improvements in diagnostics.

Despite the fact that *Aerococcus urinae* is rare organism causing infective endocarditis, most cases respond well to antibiotic theray and surgery is often not needed ^3^. The indications for surgical intervention for PVE include severe prosthetic dysfunction, severe heart failure, large vegetation, and abscess or peri-valvular involvement ^7^.

This case highlights the importance of source control by expediting prosthesis removal in the presence of overt symptoms of worsening cardiac failure and worsening prosthesis dysfunction (regurgitation in this case), as medical therapy alone may not be sufficient to effectively treat *Aerococcus urinae* IE despite appropriate sensitivities. Early identification is crucial and can be life-saving. The current diagnostic testing for microorganisms – whereas partial 16S rRNA gene sequencing analysis would be the most time-efficient method, it’s rarely done, as the expertise is limited and costs are high. Recently, there is good evidence for the use of MALDI-TOF ^8, 9^ due to increased detection rates, even in direct comparison to 16s sequencing.

In conclusion, *Aerococcus urinae* has been increasingly identified as the cause of infective endocarditis due to advancement in detection and identification methods. Therefore establishing a concise and broadly acknowledged protocol for diagnosis up to patient management is critical.

## Consent

Written informed consent for publication of their clinical details was obtained from the patient. Permission was also granted from a next of kin for publication of the manuscript.
