# Supplementary material for: Case Report: A rare case of prosthetic valve infective endocarditis caused by Aerococcus urinae
Source: F1000Res. 2018 Mar 28;6:1998. Originally published 2017 Nov 13. [Version 3] doi: 10.12688/f1000research.12776.3 (PMC5883383; doi:10.12688/f1000research.12776.3)
Supplement: Peer review history

# Peer review: Referee response for version 3

**DOI:** 10.5256/f1000research.15480.r32542

**Published:** 2018-04-03

**Authors:** Jens J Christensen

**Affiliations:** 1 Department of Clinical Microbiology, Slagelse Hospital, Slagelse, Denmark

The improvement suggestions have been taken care of and the manuscript acceptable.

I have read this submission. I believe that I have an appropriate level of expertise to confirm that it is of an acceptable scientific standard.

# Peer review: Referee response for version 2

**DOI:** 10.5256/f1000research.15259.r31034

**Published:** 2018-03-01

**Authors:** Magnus Rasmussen

**Affiliations:** 1 Department of Clinical Sciences, Division of Infection Medicine, Faculty of Medicine, Lund University, Lund, Sweden

Most concerns have been satisfactorily addressed. Information on method of species determination and on the size of the vegetation would still be of interest.

I have read this submission. I believe that I have an appropriate level of expertise to confirm that it is of an acceptable scientific standard.

# Peer review: Referee response for version 2

**DOI:** 10.5256/f1000research.15259.r31035

**Published:** 2018-02-28

**Authors:** Jens J Christensen

**Affiliations:** 1 Department of Clinical Microbiology, Slagelse Hospital, Slagelse, Denmark

Follow up on previous comments. Suggests the following improvements:

 Abstract

1. There are 2 dots ahead of Improvements: Delete 1.
2. MALDI-TOF MS has improved recognition not isolation. Please rephrase.

Introduction

1. Please rephrase “secondary to Aerococcus urinae”
2. Please give a reference on initial recognition of A. urinae. Is the year 1967 correct?
3. As can be seen in this case can be deleted.

Case report

1. Give entity on C-reactive protein
2. Blood-culture system used is missing
3. MALDI-TOF MS details are missing
4. Antibiotic susceptibility testing system used is missing

Discussion

1. The main problem… and the rest of the section: The 2 sentences are not clear. Please rephrase.
2. In conclusion….: it is advancements in as well detection as identification methods. The last sentence is not clear. Please rephrase

I have read this submission. I believe that I have an appropriate level of expertise to confirm that it is of an acceptable scientific standard, however I have significant reservations, as outlined above.

# Response

**Published:** 2018-03-05

**Authors:** Carina Dehner

**Affiliations:** Yale University, USA

Dear Dr. Christensen,

Thank you very much for your helpful feedback. We rephrased the suggested points throughout the article. 

Unfortunately we were not able to assess the points listed in the case report in our patient, however our aim was to underline the need for new techniques (like MALDI-TOF) in order to make the correct diagnosis in a timely manner.

# Peer review: Referee response for version 1

**DOI:** 10.5256/f1000research.13841.r29616

**Published:** 2018-01-22

**Authors:** Jens J Christensen

**Affiliations:** 1 Department of Clinical Microbiology, Slagelse Hospital, Slagelse, Denmark

A fatal case of IE caused by Aerococcus urinae,  in a 75-year-old man with a bioprosthetic aortic valve is presented and discussed. Very precise and covering comments have been given by reviewer 1. Microbiological data should be examined thouroughly and extended. Language correction seems indicated. The following comments can be added.

 1)      Abstract:

- It is always important to also having focus on more rare etiologies of IE. In the abstract it is stated that the mortality rate is high. This is suggested to be modified to: Initial descriptions of collections of IE cases with A. urinae demonstrated a high morbidity and mortality rate, whereas a recent Swedish epidemiological study could not retrieve this.

 

 2)      Introduction:

- Dukes criteria should be mentioned.   
- There is not a species named Streptococcus viridans.
- Recent diagnostic improvements should be included, especially MALDI-TOF mass spectrometry.
- a gram-positive, catalase-negative

  

 3)     Case description:

- Specific description of PM electrode findings should be given.
- A more detailed disease timespan is desirable.
- Microbiological data are very scarce. Blood-culture system and number of positive bottles should be given. Likewise identification criteria and susceptibility methods and results, including MIC values of relevant antibiotics should be given.
- A thorough microbiological examination of the manuscript seems indicated
- Aerococcus urinae should only be fully written the first time  

4)   Discussion

- 16S is slang: it should be partial 16S rRNA gene sequencing analysis

I have read this submission. I believe that I have an appropriate level of expertise to confirm that it is of an acceptable scientific standard, however I have significant reservations, as outlined above.

# Response

**Published:** 2018-02-13

**Authors:** Carina Dehner

**Affiliations:** Yale University, USA

1. The prognosis of *A. urinae *IE is not poor. Many cases with fatal outcome have been published but in the only population-based survey ^1^ demonstrate a relatively favourable prognosis compared to other pathogens. The risk with case reports is a publication-bias where only dramatic cases are published. The case series should be quoted and is the only reliable source on information on *A. urinae *IE. A poor prognosis is claimed in the abstract, introduction and discussion. This claim must be modified based on the findings by Sunnerhagen *et al*.

**A new reference was added to the introduction about favourable outcome to IE caused by Aerococcus urinae**

2. A diagnosis of IE is established through the Dukes criteria, I suggest a reference to Li is given ^2^.

Symptoms and chest X-ray are irrelevant for the diagnostic process. Please modify introduction.

**A new reference was added to diagnose IE based on Dukes criteria or their modifications**

3. In the case description it is twice stated that the patient has sepsis. Sepsis-3 criteria are not fulfilled. Please rephrase.

**Rephrased**

4. The cultures grew *A. urinae. *How was the species determination performed? How many cultures? MIC for ampicillin and gentamycin should be given.

**MICs for ampicillin and gentamicin added, description added about blood cultures**

5. It is claimed that TEE demonstrates a “moderate aortic regurgitation due to a large mobile vegetation”. It is important if the regurgitation was paravalvular or through the valves. Maximum size of the vegetation is also crucial since this is important for establishing the indication for operation. Left ventricular function should also be commented on as well as if there were signs of vegetations on the pacemaker cable.

**Transesophgeal echo description expanded, commented on LV and pacemaker lead.**

6. It is claimed that ampi+genta was commenced according to local guidelines. For which bacterial species are these guidelines meant. The use of aminoglycosides in this condition is controversial ^1^.

**Hospital guidelines for suspected/possible IE were followed**

7. How was “progressive aortic regurgitation verified? Could pacemaker failure have played a role?

**Repeat transthoracic echo showed worsening of regurgitation; this is added to the case**

8. Why was the patient not moved for emergency surgery when he deteriorated? This seem like an avoidable fatality!

**Deterioration was sudden and rapid, arrangements were made but patient died before the surgery**

9. In the discussion it is claimed that there are only 20 reports. This is not true ^1^. Cases up until 2015 are summarized in a review ^3^.

**Updated number of Aerococcus urinae IE  reported**

10. Surgical intervention is claimed to be common in the discussion with a quote to Wang. Please read and quote Sunnerhagen instead ^1^. Surgery is relatively rarely needed.

**Reference added to show that surgery in most cases is not indicated**

11. “Large persistent vegetation” is claimed as an indication for surgery. Large is enough.

**Modified**

12 “The presence of vegetation on the valve created a consistent source of bacteria that could embolize and can serve as a source of sepsis.” This statement has nothing to do with the current case and should be omitted.

**Omitted**

13 “The main problem is current diagnostic testing for microorganisms– whereas 16s sequencing would be the most time-efficient method, it’s rarely done, as the expertise is limited and costs… and so on” This is irrelevant for the case since the reason to operate is not dependent on microbiological diagnostics. Irrespective of the causative pathogen this patient would have been saved by timely heart surgery.

**This is kept; we wish our readers to know that improved methods of isolation are important and could help with management**

14. The claim “In conclusion, Aerococcus urinae used to be a rare cause of IE but

rates have been increasing significantly within the last 10 years.” Lacks support and should be deleted. *A. urinae* has been increasingly REPORTED as a cause of IE but incidence is likely unchanged.

**This is now added that that increase in reported cases is due to better isolation methods.**

15. In discussing Duke criteria in the case presentation one must keep in mind that *A. urinae* in 2/2 cultures (4/4 bottles) only fulfill Duke criteria if the cultures were taken with

**Agreed**

# Peer review: Referee response for version 1

**DOI:** 10.5256/f1000research.13841.r28819

**Published:** 2017-12-11

**Authors:** Magnus Rasmussen

**Affiliations:** 1 Department of Clinical Sciences, Division of Infection Medicine, Faculty of Medicine, Lund University, Lund, Sweden

This work describes a case of prosthetic valve infective (IE) caused by Aerococcus urinae with fatal outcome. The number of case reports on this condition is increasing and it is not immediately obvious that another case with poor outcome is helpful. This case, however, has an important learning point in that a patient with prosthetic valve endocarditis, in resource-rich settings, must be treated in a centre where acute cardiac surgery can be performed or near such a centre. It is of less importance if the causative bacterium in this case were A. urinae or any other bacterium. I list my major concerns and minor points below:

 Major concerns

 1. The prognosis of *A. urinae *IE is not poor. Many cases with fatal outcome have been published but in the only population-based survey ^1^ demonstrate a relatively favourable prognosis compared to other pathogens. The risk with case reports is a publication-bias where only dramatic cases are published. The case series should be quoted and is the only reliable source on information on *A. urinae *IE. A poor prognosis is claimed in the abstract, introduction and discussion. This claim must be modified based on the findings by Sunnerhagen *et al*.

 2. A diagnosis of IE is established through the Dukes criteria, I suggest a reference to Li is given ^2^. Symptoms and chest X-ray are irrelevant for the diagnostic process. Please modify introduction.

 3. In the case description it is twice stated that the patient has sepsis. Sepsis-3 criteria are not fulfilled. Please rephrase.

 4. The cultures grew *A. urinae. *How was the species determination performed? How many cultures? MIC for ampicillin and gentamycin should be given.

 5. It is claimed that TEE demonstrates a “moderate aortic regurgitation due to a large mobile vegetation”. It is important if the regurgitation was paravalvular or through the valves. Maximum size of the vegetation is also crucial since this is important for establishing the indication for operation. Left ventricular function should also be commented on as well as if there were signs of vegetations on the pacemaker cable.

 6. It is claimed that ampi+genta was commenced according to local guidelines. For which bacterial species are these guidelines meant. The use of aminoglycosides in this condition is controversial ^1^.

 7. How was “progressive aortic regurgitation verified? Could pacemaker failure have played a role?

 8. Why was the patient not moved for emergency surgery when he deteriorated? This seem like an avoidable fatality!

 9. In the discussion it is claimed that there are only 20 reports. This is not true ^1^. Cases up until 2015 are summarized in a review ^3^.

 10. Surgical intervention is claimed to be common in the discussion with a quote to Wang. Please read and quote Sunnerhagen instead ^1^. Surgery is relatively rarely needed.

 11. “Large persistent vegetation” is claimed as an indication for surgery. Large is enough.

 12 “The presence of vegetation on the valve created a consistent source of bacteria that could embolize and can serve as a source of sepsis.” This statement has nothing to do with the current case and should be omitted.

 13 “The main problem is current diagnostic testing for microorganisms– whereas 16s sequencing would be the most time-efficient method, it’s rarely done, as the expertise is limited and costs… and so on” This is irrelevant for the case since the reason to operate is not dependent on microbiological diagnostics. Irrespective of the causative pathogen this patient would have been saved by timely heart surgery.

 14. The claim “In conclusion, Aerococcus urinae used to be a rare cause of IE but

 rates have been increasing significantly within the last 10 years.” Lacks support and should be deleted. *A. urinae* has been increasingly REPORTED as a cause of IE but incidence is likely unchanged.

 15. In discussing Duke criteria in the case presentation one must keep in mind that *A. urinae* in 2/2 cultures (4/4 bottles) only fulfill Duke criteria if the cultures were taken with

 Minor comments

 1. I suggest another title. Something like “fatal case of A. urinae prosthetic valve endocrditis.”

 2. Why mention HACEK in the abstract? Those organisms are exceedingly rare and for example much less common than betaheamolytic strep.

 3. In case presentation spell out JVD.

 4. “Stable haemoglobin”- what is meant. Are the authors referring to repeated measurements?

I have read this submission. I believe that I have an appropriate level of expertise to confirm that it is of an acceptable scientific standard, however I have significant reservations, as outlined above.

## References

[1] SunnerhagenTNilsonBOlaisonLRasmussenM: Clinical and microbiological features of infective endocarditis caused by aerococci. *Infection*.2016;44(2) : 10.1007/s15010-015-0812-8 167-73 10.1007/s15010-015-0812-8 26119199

[2] LiJSSextonDJMickNNettlesRFowlerVGRyanTBashoreTCoreyGR: Proposed modifications to the Duke criteria for the diagnosis of infective endocarditis. *Clin Infect Dis*.2000;30(4) : 10.1086/313753 633-8 10.1086/313753 10770721

[3] RasmussenM: Aerococcus: an increasingly acknowledged human pathogen. *Clin Microbiol Infect*.2016;22(1) : 10.1016/j.cmi.2015.09.026 22-27 10.1016/j.cmi.2015.09.026 26454061

# Response

**Published:** 2018-02-13

**Authors:** Carina Dehner

**Affiliations:** Yale University, USA

1)      Abstract:

-     It is always important to also having focus on more rare etiologies of IE. In the abstract it is stated that the mortality rate is high. This is suggested to be modified to: Initial descriptions of collections of IE cases with A. urinae demonstrated a high morbidity and mortality rate, whereas a recent Swedish epidemiological study could not retrieve this.

**New reference to the introduction was added to highlight the better outcome**

2)      Introduction:

-      Dukes criteria should be mentioned.   

**- New references added to mention Duke’s criteria or its modifications**

-      There is not a species named Streptococcus viridans.

**Correction made**

·       Recent diagnostic improvements should be included, especially MALDI-TOF mass spectrometry.

**Added in abstract**

-      a gram-positive, catalase-negative

**Correction made**

  

3)     Case description: Specific description of PM electrode findings should be given.

**Included in description, PM lead was not involved.**

Microbiological data are very scarce. Blood-culture system and number of positive bottles should be given. Likewise identification criteria and susceptibility methods and results, including MIC values of relevant antibiotics should be given.

**This is now added to the case description**

4)   Discussion: 16S is slang: it should be partial 16S rRNA gene sequencing analysis

**Correction made**
